# DISCRIMINATION IS ASSOCIATED WITH HIGHER ODDS OF HOSPITALIZATIONS AMONG OLDER AFRICAN AMERICANS

**DOI:** 10.1093/geroni/igad104.0513

**Published:** 2023-12-21

**Authors:** Brittney Lange Maia, Bryan James, Ana Capuano, Francine Grodstein, Lisa Barnes

**Affiliations:** Rush Alzheimer’s Disease Center, Chicago, Illinois, United States; Rush University Medical Center, Chicago, Illinois, United States; Rush University Medical Center, Chicago, Illinois, United States; Rush Alzheimer’s Disease Center, Chicago, Illinois, United States; Rush University Medical Center, Chicago, Illinois, United States

## Abstract

Everyday discrimination—experiences of being treated unfairly based on background characteristics like race—is linked to poor physical and mental health throughout the lifespan. Whether more experiences of discrimination are associated with higher likelihood of being hospitalized in older African Americans has not been explored. Hospitalization can represent both poor health and healthcare access. Participants were community-dwelling African Americans from the Rush Memory and Aging Project or Minority Aging Research Study, longitudinal studies of aging (N=301 with at least 12 months linked Medicare fee-for-service claims; mean age 72.5 years (standard deviation [SD]: 5.7), 79% female). Discrimination was assessed using the Detroit Area Study Everyday Discrimination Scale. Hospitalizations (sub-categorized as elective/non-elective, surgical) were quantified using Medicare claims. Mixed-effects ordinal logistic regression models tested associations between baseline discrimination and subsequent odds of hospitalizations per year (0, 1, 2+). The mean baseline discrimination score was 1.7 (SD: 2.2). Over an average 6.5 years (SD: 4.1), 160 participants had at least 1 hospitalization (respectively, 118, 87, and 127 participants had at least 1 nonelective, elective, or surgical hospitalization). Adjusting for age, sex, education, income, depressive symptoms, and medical comorbidity, more experiences of discrimination were associated with higher odds of hospitalization (odds ratio [OR] per point higher on discrimination score=1.12, 95% CI: 1.03-1.22), and higher odds of nonelective (OR=1.12, 95% CI: 1.01-1.24), but not surgical or elective hospitalizations. Drivers of these associations, which may include preventive healthcare avoidance due to discrimination or poor health due to the chronic stress response to discrimination, should be explored.

